# Steroid-Induced Sinus Bradycardia

**DOI:** 10.7759/cureus.15065

**Published:** 2021-05-16

**Authors:** Kanika Khandelwal, Rajasekhar R Madathala, Nikhita Chennaiahgari, Mohammed Yousuffuddin

**Affiliations:** 1 Internal Medicine, Mayo Clinic Health System, Austin, USA; 2 Hepatology and Transplant, Mayo Clinic, Rochester, USA

**Keywords:** prednisone, bradycardia

## Abstract

Steroids are one of the most commonly used drugs and known to be associated with several side effects. There have been case reports about the associated sinus bradycardia with pulse dose corticosteroids administration both IV and oral. We present a case of asymptomatic sinus bradycardia associated with oral prednisone 40 mg. A 69-year-old male was admitted to the ICU for sepsis and subsequently was found to have gastrointestinal (GI) bleed. He developed an acute gout attack during hospitalization and was treated with prednisone 40 mg. Over the next 24 hours, the patient's heart rate dropped to 30s to 40s beats/minute while other vitals have remained stable. He was monitored on telemetry and review of the rhythm strips, as well as a 12-lead electrocardiogram (EKG), that showed sinus bradycardia; no pauses or atrio-ventricular (AV) nodal blocks were identified. The patient was not on any beta blocker or other therapies commonly associated with sinus bradycardia. His steroids were stopped while all other medications were continued. His heart rate slowly started to improve over the next 24 hours. He was not found to have any further episodes of bradycardia. Our case is unusual as we noted transient asymptomatic bradycardia with oral prednisone 40 mg dose. While bradycardia is reversible and may go unnoticed, it is important for the clinician to be aware of this adverse effect and include it in the list of potential differentials for bradycardia.

## Introduction

Steroids are one of the most commonly used drugs in hospital and outpatient setting for a wide variety of pathologies ranging from chronic obstructive pulmonary disease (COPD)/asthma exacerbation to autoimmune diseases and inflammatory conditions. Common complications associated with steroids including hyperglycemia, electrolyte abnormalities, hypertension, delirium, and behavioral changes in elderly patients; serious adverse events including cardiac arrhythmias have been reported in the literature [1-3]. Over the years, few case reports have been published about the associated sinus bradycardia with pulse dose corticosteroids administration both IV and oral [1-2,4]. We report a case of asymptomatic sinus bradycardia associated with once-daily dose of 40 mg oral prednisone.

## Case presentation

**Figure 1:**
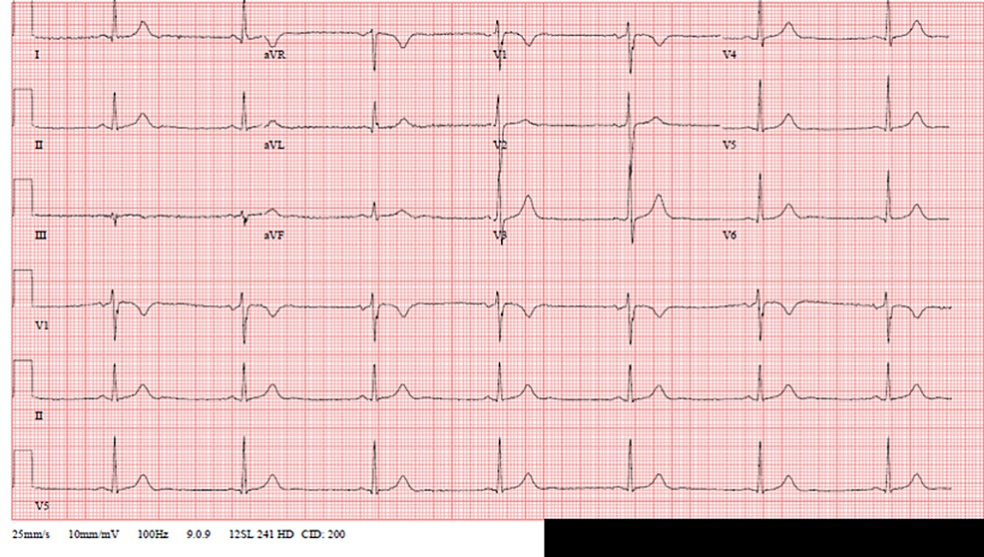
Electrocardiogram-sinus bradycardia

This is a 69-year-old male who presented to the urology clinic with fever, chills, and bilateral flank pain. A day before, he underwent bilateral ureteroscopy for a left ureteropelvic junction (UPJ) stone and bilateral nonobstructing nephrolithiasis. His past medical history includes hyperlipidemia on statins, gout (not on any medications), and bicuspid aortic valve. He was found to be febrile, tachycardic, and hypotensive in the clinic; he was then admitted to the hospital for sepsis secondary to urinary tract infection (UTI) (admission vitals included in Table 1). His blood pressure improved with IV fluid resuscitation and he was started on IV piperacillin and tazobactam after obtaining blood and urine cultures. His blood and urine cultures returned positive for Escherichia coli (resistant to Bactrim, sensitive to ceftriaxone), hence, antibiotics were narrowed down to ceftriaxone. On day 4 of hospitalization, the patient started complaining of epigastric abdominal discomfort with occasional black stools. He was started on pantoprazole 40 mg twice daily and serial hemoglobin was monitored which remained stable. On hospital day 8, he complained of bilateral calf and great toe pain. Doppler ultrasound of the lower extremity veins was obtained which ruled out deep vein thrombosis (DVT). Given his history of gout, uric acid level was obtained which was normal; the patient was empirically started on prednisone 40 mg PO daily with dramatic improvement in his lower extremity pain. Given his abdominal discomfort, nonsteroidal anti-inflammatory drugs (NSAIDs) and colchicine were avoided. Over the next 24 hours, the patient's heart rate dropped to 30s to 40s beats/minute while other vitals remained stable. No hypoxia was noted during this time. He has monitored on the telemetry and a review of the rhythm strips showed sinus bradycardia; no pauses or atrio-ventricular (AV) nodal blocks were identified. A 12-lead electrocardiogram (EKG) was obtained which showed sinus bradycardia without any other arrhythmias (Figure 1).

**Table 1:** Sequence of events Heart rate preceding and following the administration of prednisone HR: heart rate; SBP: systolic blood pressure; UTI: urinary tract infection; EKG: electrocardiogram; GI: gastrointestinal; E.Coli: Escherichia coli.

Day 0 (Elective procedure)		Ureteroscopy with laser lithotripsy bilateral for nephrolithiasis
Day 1 (Clinic)	Temp 39.3, HR 109, SBP 92/51	IV fluids administered
Day 1 (Admission to hospital)	BP: 128/64, HR: 105 admitted for sepsis secondary to UTI	IV Piperacillin/Tazobatum initiated
Day 2	HR: 80s, Blood cultures positive for E.Coli	IV Piperacillin/tazobactum discontinued, started IV ceftriaxone
Day 4	HR: 60s, Stool hemoccult positive	Pantoprazole 40 mg bid started for possible Upper GI bleed.
Day 8	HR: 60s, Toe pain (Acute gout attack)	Prednisone 40 mg initiated (Ist dose of prednisone)
Day 9	HR: 30s-40s, Black stools, normotensive	2nd and last dose of prednisone administered, all other medications continued.
Day 10, morning	HR: 30s-40s, EKG showed sinus bradycardia. Patient asymptomatic	Prednisone discontinued, all other medications were continued.
Day 10, afternoon	HR: 50, Endoscopy showed: nonbleeding duodenal ulcers	
Day 10, later during the day	HR: 60s, O2 saturation in 70s, cyanosis; methemoglobin >30	Methylene blue administered, improvement in oxygenation as well as cyanosis observed.
Day 11	HR: 60s	Patient discharged home with 48 Holter monitor
Day 24	HR: 60s	Follow up with cardiology in the clinic. No bradycardia noted, no other etiology of bradycardia noted.

Thyroid level was normal, patient was not on any beta blocker or other therapies commonly associated with sinus bradycardia. The patient remained asymptomatic during this time.

After a review of the literature for bradycardia, his steroids were stopped while all other medications were continued. His heart rate slowly started to improve over the next 24 hours.

The next day patient underwent endoscopy for melena and was found to have a nonbleeding duodenal ulcer. Immediately after he returned from the endoscopy, he had a fainting spell and was noted to be hypoxic with oxygen saturation in the ’70s. He remained hypoxic to 80s even on 100% oxygen, X-ray of the chest did not reveal any acute finding. EKG revealed normal sinus rhythm with heart rate around 60 beats/minute; arterial blood gas revealed the patient had a methemoglobin level of 30. The patient was immediately treated with methylene blue 1mg/kg with dramatic improvement in his hypoxia. A review of his medications revealed he had received cetacaine spray during endoscopy, which was attributed to his methemoglobinemia. He was observed overnight and subsequently discharged home the next day. Holter monitor obtained as outpatient showed normal sinus rhythm without any episodes of bradycardia.

## Discussion

Corticosteroids are widely used in our current clinical practice both in the inpatient and outpatient setting. Common complications of steroids are well documented in literature including hypertension, hyperglycemia, electrolyte abnormality, and delirium in elderly patients, but very little is known about the cardiac arrhythmias in patients using steroids. There are few case reports over the years about the association of pulse dose corticosteroid therapy and cardiac arrhythmias especially sinus bradycardia [1-2,5]. In most of the case reports, the patient either received IV dexamethasone [4,6] or IV methylprednisolone [4-5,7] or high dose oral prednisolone [8]. There is one study where sinus bradycardia was documented after oral methylprednisolone dose of 52 mg as well as with tapering dose of prednisone [3]. Wide variety of arrhythmias including ventricular ectopic, ventricular tachycardias and sinus tachycardia, and bradycardias [1-2] are identified, in a prospective trial done by Jain et al. [1]; 33% of patient have been noted to develop sinus bradycardia. In most of the case reports, the patient had asymptomatic sinus bradycardia and heart rate improved with tapering or stopping steroids.

In our case, bradycardia resolved after discontinuation of prednisone. The Naranjo scale is a tool used to estimate the probability of an adverse drug event, with scores ranging from 0 to 13 and a higher score suggesting a stronger probability [9]. Using this scale, we calculated a score of 7 which suggests probable reaction (Table 2).

**Table 2:** Naranjo adverse reaction probability scale

Questions	Yes	No	Do not know	Score
Are there previous conclusive reports on this reaction?	+1	0	0	+1
Did the adverse event appear after the suspected drug was administered?	+2	-1	0	+2
Did the adverse reaction improve when the drug was discontinued, or a specific antagonist was administered?	+1	0	0	+1
Did the adverse event reappear when the drug was readministered?	+2	-1	0	0
Are there alternative causes (other than the drug) that could on their own have caused the reaction?	-1	+2	0	+2
Did the reaction occur when the placebo was given?	-1	+1	0	0
Was the drug detected in the blood (or other fluids) in concentrations known to be toxic?	+1	0	0	0
Was the reaction more severe when the dose was increased or less severe when the dose was decreased?	+1	0	0	0
Did the patient have a similar reaction to the same or similar drugs in any previous exposure?	+1	0	0	0
Was the adverse event confirmed by any objective evidence?	+1	0	0	+1
Total score				7

Several hypotheses for steroid-induced bradycardia have been suggested, however, the exact mechanism by which this occurs is currently unknown [6]. Steroids may exert a bradycardic effect through suppression of cytokine production and function of the sympathetic nervous system [3]. This could be the possible mechanism of bradycardia in our case.

To the best of our knowledge, there was no case report in patients receiving once-daily oral prednisone at a dose of 40 mg which is commonly used dose to treat a variety of conditions including COPD/asthma. With our case report, we would like to highlight uncommon but potential side effects of sinus bradycardia in patients while using oral steroids and to remain vigilant about this possible adverse effect. 

## Conclusions

Steroids induced bradycardia is a rare phenomenon and has been reported with the use of pulse dose steroid therapy. Our case is unusual as we noted transient asymptomatic bradycardia with once-daily dose of 40 mg prednisone which is used commonly for a variety of clinical conditions. While bradycardia is reversible and may go unnoticed, it is important for the clinician to be aware of this adverse effect and include it in the list of potential differentials for bradycardia.
